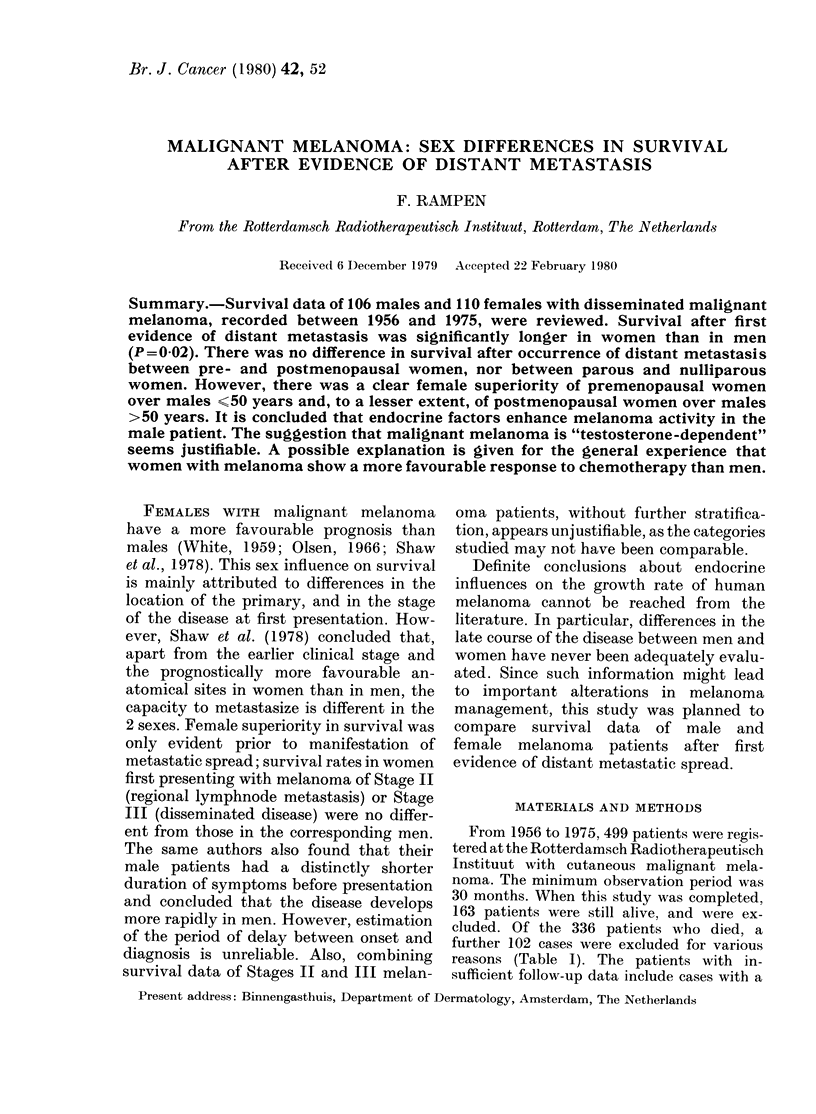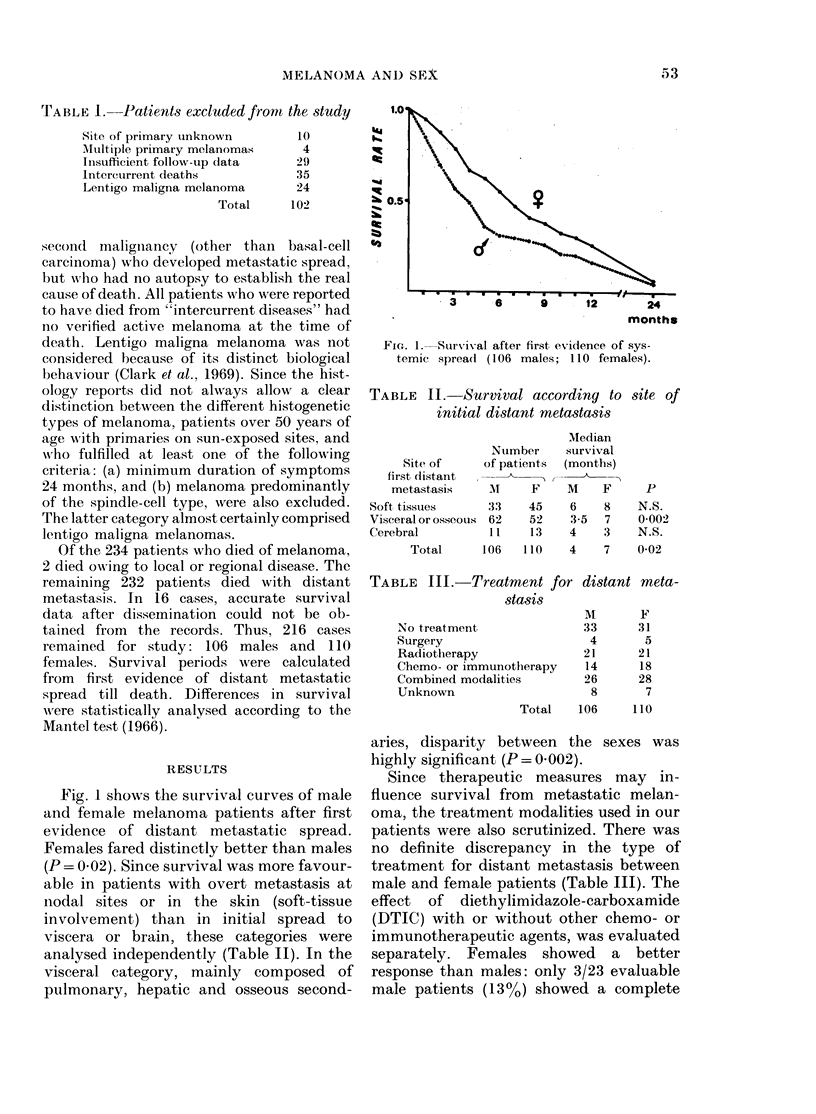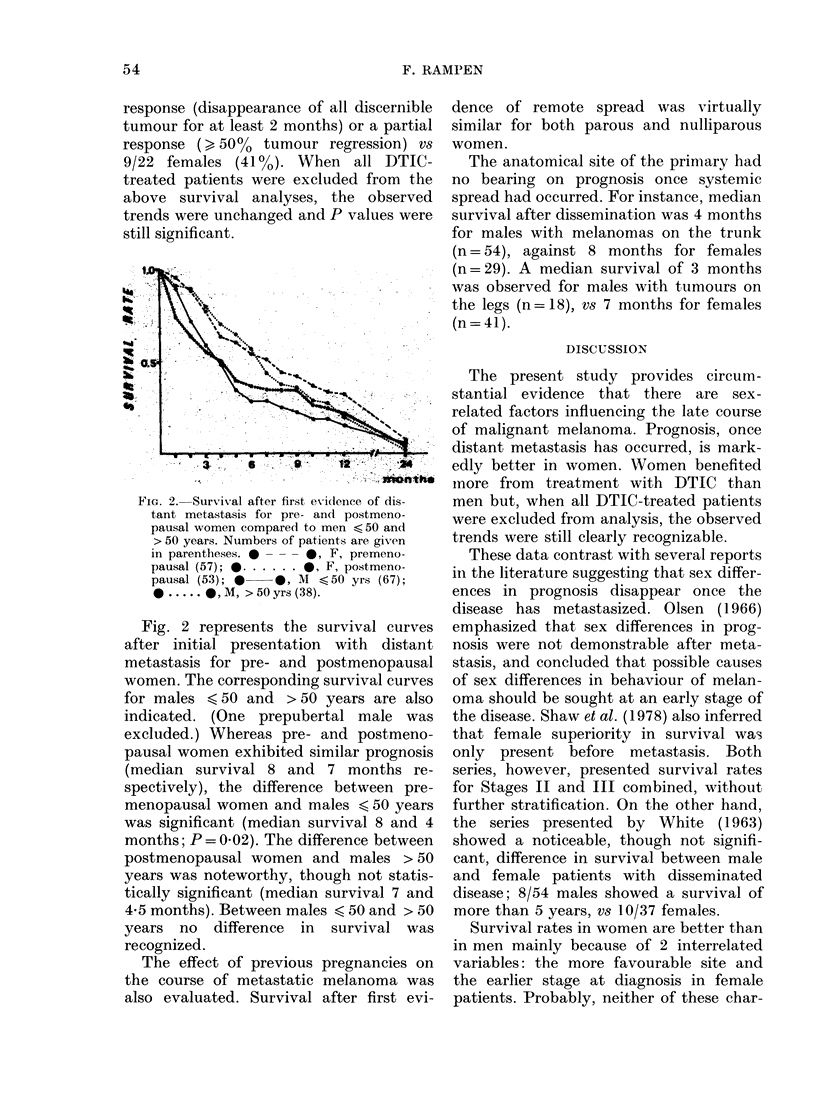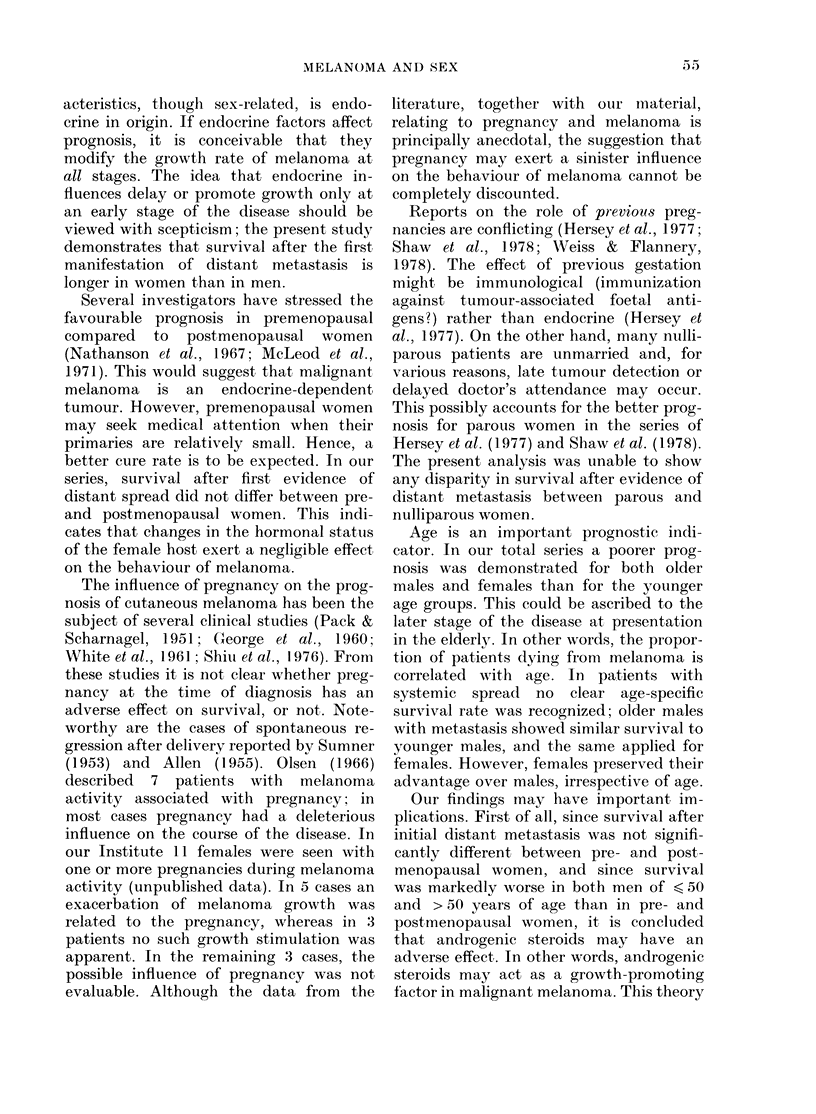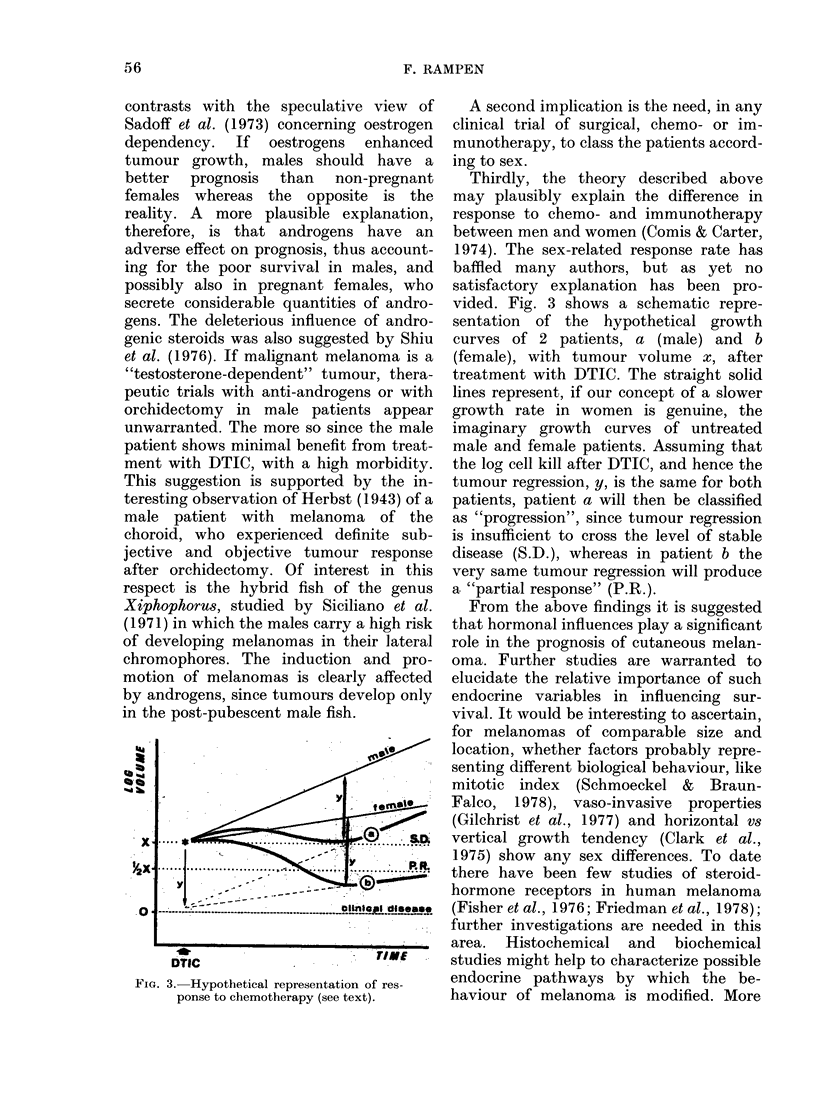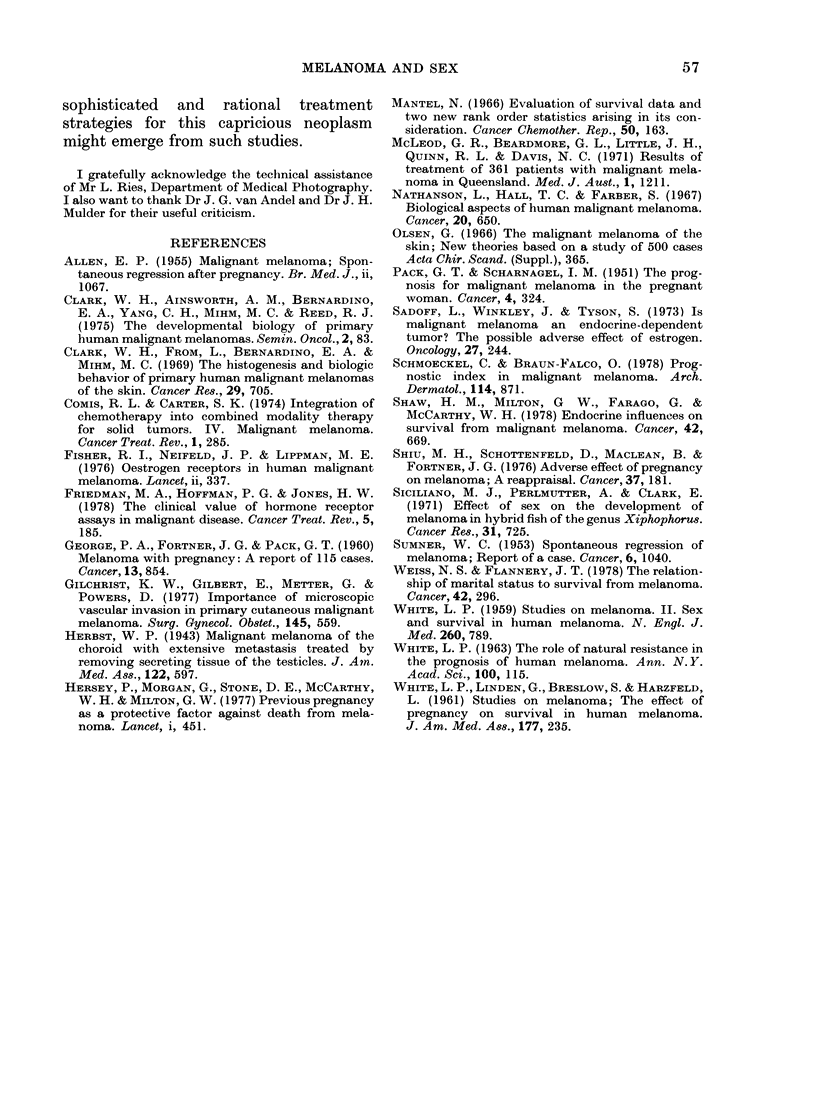# Malignant melanoma: sex differences in survival after evidence of distant metastasis.

**DOI:** 10.1038/bjc.1980.202

**Published:** 1980-07

**Authors:** F. Rampen

## Abstract

Survival data of 106 males and 110 females with disseminated malignant melanoma, recorded between 1956 and 1975, were reviewed. Survival after first evidence of distant metastasis was significantly longer in women than in men (P = 0.02). There was no difference in survival after occurrence of distant metastasis between pre- and postmenopausal women, nor between parous and nulliparous women. However, there was a clear female superiority of premenopausal women over males less than or equal to 50 years and, to a lesser extent, of postmenopausal women over males > 50 years. It is concluded that endocrine factors enhance melanoma activity in the male patient. The suggestion that malignant melanoma is "testosterone-dependent" seems justifiable. A possible explanation is given for the general experience that women with melanoma show a more favourable response to chemotherapy than men.


*Br. J. Cancer* (1980) **42,** 52

### MALIGNANT MELANOMA: SEX DIFFERENCES IN SURVIVAL AFTER EVIDENCE OF DISTANT METASTASIS

F. RAMPEN

*From the Rotterdamsch Radiotherapeutisch Instituut, Rotterdam, The Netherlands*

Received 6 December 1979 Accepted 22 February 1980

**Summary.—Survival data of 106 males and 110 females with disseminated malignant melanoma, recorded between 1956 and 1975, were reviewed. Survival after first evidence of distant metastasis was significantly longer in women than in men ($P = 0{\cdot}02$). There was no difference in survival after occurrence of distant metastasis between pre- and postmenopausal women, nor between parous and nulliparous women. However, there was a clear female superiority of premenopausal women over males $\leqslant 50$ years and, to a lesser extent, of postmenopausal women over males $> 50$ years. It is concluded that endocrine factors enhance melanoma activity in the male patient. The suggestion that malignant melanoma is "testosterone-dependent" seems justifiable. A possible explanation is given for the general experience that women with melanoma show a more favourable response to chemotherapy than men.**

FEMALES WITH malignant melanoma have a more favourable prognosis than males (White, 1959; Olsen, 1966; Shaw *et al.*, 1978). This sex influence on survival is mainly attributed to differences in the location of the primary, and in the stage of the disease at first presentation. However, Shaw *et al.* (1978) concluded that, apart from the earlier clinical stage and the prognostically more favourable anatomical sites in women than in men, the capacity to metastasize is different in the 2 sexes. Female superiority in survival was only evident prior to manifestation of metastatic spread; survival rates in women first presenting with melanoma of Stage II (regional lymphnode metastasis) or Stage III (disseminated disease) were no different from those in the corresponding men. The same authors also found that their male patients had a distinctly shorter duration of symptoms before presentation and concluded that the disease develops more rapidly in men. However, estimation of the period of delay between onset and diagnosis is unreliable. Also, combining survival data of Stages II and III melanoma patients, without further stratification, appears unjustifiable, as the categories studied may not have been comparable.

Definite conclusions about endocrine influences on the growth rate of human melanoma cannot be reached from the literature. In particular, differences in the late course of the disease between men and women have never been adequately evaluated. Since such information might lead to important alterations in melanoma management, this study was planned to compare survival data of male and female melanoma patients after first evidence of distant metastatic spread.

### MATERIALS AND METHODS

From 1956 to 1975, 499 patients were registered at the Rotterdamsch Radiotherapeutisch Instituut with cutaneous malignant melanoma. The minimum observation period was 30 months. When this study was completed, 163 patients were still alive, and were excluded. Of the 336 patients who died, a further 102 cases were excluded for various reasons (Table I). The patients with insufficient follow-up data include cases with a

Present address: Binnengasthuis, Department of Dermatology, Amsterdam, The Netherlands

TABLE I.—*Patients excluded from the study*

| | |
|---|---|
| Site of primary unknown | 10 |
| Multiple primary melanomas | 4 |
| Insufficient follow-up data | 29 |
| Intercurrent deaths | 35 |
| Lentigo maligna melanoma | 24 |
| Total | 102 |

second malignancy (other than basal-cell carcinoma) who developed metastatic spread, but who had no autopsy to establish the real cause of death. All patients who were reported to have died from "intercurrent diseases" had no verified active melanoma at the time of death. Lentigo maligna melanoma was not considered because of its distinct biological behaviour (Clark *et al.*, 1969). Since the histology reports did not always allow a clear distinction between the different histogenetic types of melanoma, patients over 50 years of age with primaries on sun-exposed sites, and who fulfilled at least one of the following criteria: (a) minimum duration of symptoms 24 months, and (b) melanoma predominantly of the spindle-cell type, were also excluded. The latter category almost certainly comprised lentigo maligna melanomas.

Of the 234 patients who died of melanoma, 2 died owing to local or regional disease. The remaining 232 patients died with distant metastasis. In 16 cases, accurate survival data after dissemination could not be obtained from the records. Thus, 216 cases remained for study: 106 males and 110 females. Survival periods were calculated from first evidence of distant metastatic spread till death. Differences in survival were statistically analysed according to the Mantel test (1966).

### RESULTS

Fig. 1 shows the survival curves of male and female melanoma patients after first evidence of distant metastatic spread. Females fared distinctly better than males ($P = 0{\cdot}02$). Since survival was more favourable in patients with overt metastasis at nodal sites or in the skin (soft-tissue involvement) than in initial spread to viscera or brain, these categories were analysed independently (Table II). In the visceral category, mainly composed of pulmonary, hepatic and osseous secondaries, disparity between the sexes was highly significant ($P = 0{\cdot}002$).

FIG. 1.—Survival after first evidence of systemic spread (106 males; 110 females).

TABLE II.—*Survival according to site of initial distant metastasis*

| Site of first distant metastasis | Number of patients M | Number of patients F | Median survival (months) M | Median survival (months) F | *P* |
|---|---|---|---|---|---|
| Soft tissues | 33 | 45 | 6 | 8 | N.S. |
| Visceral or osseous | 62 | 52 | 3·5 | 7 | 0·002 |
| Cerebral | 11 | 13 | 4 | 3 | N.S. |
| Total | 106 | 110 | 4 | 7 | 0·02 |

TABLE III.—*Treatment for distant metastasis*

| | M | F |
|---|---|---|
| No treatment | 33 | 31 |
| Surgery | 4 | 5 |
| Radiotherapy | 21 | 21 |
| Chemo- or immunotherapy | 14 | 18 |
| Combined modalities | 26 | 28 |
| Unknown | 8 | 7 |
| Total | 106 | 110 |

Since therapeutic measures may influence survival from metastatic melanoma, the treatment modalities used in our patients were also scrutinized. There was no definite discrepancy in the type of treatment for distant metastasis between male and female patients (Table III). The effect of diethylimidazole-carboxamide (DTIC) with or without other chemo- or immunotherapeutic agents, was evaluated separately. Females showed a better response than males: only 3/23 evaluable male patients (13%) showed a complete response (disappearance of all discernible tumour for at least 2 months) or a partial response (≥ 50% tumour regression) *vs* 9/22 females (41%). When all DTIC-treated patients were excluded from the above survival analyses, the observed trends were unchanged and $P$ values were still significant.

FIG. 2.—Survival after first evidence of distant metastasis for pre- and postmenopausal women compared to men ≤ 50 and > 50 years. Numbers of patients are given in parentheses. ● – – – ●, F, premenopausal (57); ●. . . . . . ●, F, postmenopausal (53); ●——●, M ≤ 50 yrs (67); ● . . . . . ●, M, > 50 yrs (38).

Fig. 2 represents the survival curves after initial presentation with distant metastasis for pre- and postmenopausal women. The corresponding survival curves for males ≤ 50 and > 50 years are also indicated. (One prepubertal male was excluded.) Whereas pre- and postmenopausal women exhibited similar prognosis (median survival 8 and 7 months respectively), the difference between premenopausal women and males ≤ 50 years was significant (median survival 8 and 4 months; $P = 0{\cdot}02$). The difference between postmenopausal women and males > 50 years was noteworthy, though not statistically significant (median survival 7 and 4·5 months). Between males ≤ 50 and > 50 years no difference in survival was recognized.

The effect of previous pregnancies on the course of metastatic melanoma was also evaluated. Survival after first evidence of remote spread was virtually similar for both parous and nulliparous women.

The anatomical site of the primary had no bearing on prognosis once systemic spread had occurred. For instance, median survival after dissemination was 4 months for males with melanomas on the trunk (n = 54), against 8 months for females (n = 29). A median survival of 3 months was observed for males with tumours on the legs (n = 18), *vs* 7 months for females (n = 41).

### DISCUSSION

The present study provides circumstantial evidence that there are sex-related factors influencing the late course of malignant melanoma. Prognosis, once distant metastasis has occurred, is markedly better in women. Women benefited more from treatment with DTIC than men but, when all DTIC-treated patients were excluded from analysis, the observed trends were still clearly recognizable.

These data contrast with several reports in the literature suggesting that sex differences in prognosis disappear once the disease has metastasized. Olsen (1966) emphasized that sex differences in prognosis were not demonstrable after metastasis, and concluded that possible causes of sex differences in behaviour of melanoma should be sought at an early stage of the disease. Shaw *et al.* (1978) also inferred that female superiority in survival was only present before metastasis. Both series, however, presented survival rates for Stages II and III combined, without further stratification. On the other hand, the series presented by White (1963) showed a noticeable, though not significant, difference in survival between male and female patients with disseminated disease; 8/54 males showed a survival of more than 5 years, *vs* 10/37 females.

Survival rates in women are better than in men mainly because of 2 interrelated variables: the more favourable site and the earlier stage at diagnosis in female patients. Probably, neither of these characteristics, though sex-related, is endocrine in origin. If endocrine factors affect prognosis, it is conceivable that they modify the growth rate of melanoma at *all* stages. The idea that endocrine influences delay or promote growth only at an early stage of the disease should be viewed with scepticism; the present study demonstrates that survival after the first manifestation of distant metastasis is longer in women than in men.

Several investigators have stressed the favourable prognosis in premenopausal compared to postmenopausal women (Nathanson *et al.*, 1967; McLeod *et al.*, 1971). This would suggest that malignant melanoma is an endocrine-dependent tumour. However, premenopausal women may seek medical attention when their primaries are relatively small. Hence, a better cure rate is to be expected. In our series, survival after first evidence of distant spread did not differ between pre- and postmenopausal women. This indicates that changes in the hormonal status of the female host exert a negligible effect on the behaviour of melanoma.

The influence of pregnancy on the prognosis of cutaneous melanoma has been the subject of several clinical studies (Pack & Scharnagel, 1951; George *et al.*, 1960; White *et al.*, 1961; Shiu *et al.*, 1976). From these studies it is not clear whether pregnancy at the time of diagnosis has an adverse effect on survival, or not. Noteworthy are the cases of spontaneous regression after delivery reported by Sumner (1953) and Allen (1955). Olsen (1966) described 7 patients with melanoma activity associated with pregnancy; in most cases pregnancy had a deleterious influence on the course of the disease. In our Institute 11 females were seen with one or more pregnancies during melanoma activity (unpublished data). In 5 cases an exacerbation of melanoma growth was related to the pregnancy, whereas in 3 patients no such growth stimulation was apparent. In the remaining 3 cases, the possible influence of pregnancy was not evaluable. Although the data from the literature, together with our material, relating to pregnancy and melanoma is principally anecdotal, the suggestion that pregnancy may exert a sinister influence on the behaviour of melanoma cannot be completely discounted.

Reports on the role of *previous* pregnancies are conflicting (Hersey *et al.*, 1977; Shaw *et al.*, 1978; Weiss & Flannery, 1978). The effect of previous gestation might be immunological (immunization against tumour-associated foetal antigens?) rather than endocrine (Hersey *et al.*, 1977). On the other hand, many nulliparous patients are unmarried and, for various reasons, late tumour detection or delayed doctor's attendance may occur. This possibly accounts for the better prognosis for parous women in the series of Hersey *et al.* (1977) and Shaw *et al.* (1978). The present analysis was unable to show any disparity in survival after evidence of distant metastasis between parous and nulliparous women.

Age is an important prognostic indicator. In our total series a poorer prognosis was demonstrated for both older males and females than for the younger age groups. This could be ascribed to the later stage of the disease at presentation in the elderly. In other words, the proportion of patients dying from melanoma is correlated with age. In patients with systemic spread no clear age-specific survival rate was recognized; older males with metastasis showed similar survival to younger males, and the same applied for females. However, females preserved their advantage over males, irrespective of age.

Our findings may have important implications. First of all, since survival after initial distant metastasis was not significantly different between pre- and postmenopausal women, and since survival was markedly worse in both men of $\leqslant 50$ and $>50$ years of age than in pre- and postmenopausal women, it is concluded that androgenic steroids may have an adverse effect. In other words, androgenic steroids may act as a growth-promoting factor in malignant melanoma. This theory contrasts with the speculative view of Sadoff *et al.* (1973) concerning oestrogen dependency. If oestrogens enhanced tumour growth, males should have a better prognosis than non-pregnant females whereas the opposite is the reality. A more plausible explanation, therefore, is that androgens have an adverse effect on prognosis, thus accounting for the poor survival in males, and possibly also in pregnant females, who secrete considerable quantities of androgens. The deleterious influence of androgenic steroids was also suggested by Shiu *et al.* (1976). If malignant melanoma is a "testosterone-dependent" tumour, therapeutic trials with anti-androgens or with orchidectomy in male patients appear unwarranted. The more so since the male patient shows minimal benefit from treatment with DTIC, with a high morbidity. This suggestion is supported by the interesting observation of Herbst (1943) of a male patient with melanoma of the choroid, who experienced definite subjective and objective tumour response after orchidectomy. Of interest in this respect is the hybrid fish of the genus *Xiphophorus*, studied by Siciliano *et al.* (1971) in which the males carry a high risk of developing melanomas in their lateral chromophores. The induction and promotion of melanomas is clearly affected by androgens, since tumours develop only in the post-pubescent male fish.

FIG. 3.—Hypothetical representation of response to chemotherapy (see text).

A second implication is the need, in any clinical trial of surgical, chemo- or immunotherapy, to class the patients according to sex.

Thirdly, the theory described above may plausibly explain the difference in response to chemo- and immunotherapy between men and women (Comis & Carter, 1974). The sex-related response rate has baffled many authors, but as yet no satisfactory explanation has been provided. Fig. 3 shows a schematic representation of the hypothetical growth curves of 2 patients, *a* (male) and *b* (female), with tumour volume *x*, after treatment with DTIC. The straight solid lines represent, if our concept of a slower growth rate in women is genuine, the imaginary growth curves of untreated male and female patients. Assuming that the log cell kill after DTIC, and hence the tumour regression, *y*, is the same for both patients, patient *a* will then be classified as "progression", since tumour regression is insufficient to cross the level of stable disease (S.D.), whereas in patient *b* the very same tumour regression will produce a "partial response" (P.R.).

From the above findings it is suggested that hormonal influences play a significant role in the prognosis of cutaneous melanoma. Further studies are warranted to elucidate the relative importance of such endocrine variables in influencing survival. It would be interesting to ascertain, for melanomas of comparable size and location, whether factors probably representing different biological behaviour, like mitotic index (Schmoeckel & Braun-Falco, 1978), vaso-invasive properties (Gilchrist *et al.*, 1977) and horizontal *vs* vertical growth tendency (Clark *et al.*, 1975) show any sex differences. To date there have been few studies of steroid-hormone receptors in human melanoma (Fisher *et al.*, 1976; Friedman *et al.*, 1978); further investigations are needed in this area. Histochemical and biochemical studies might help to characterize possible endocrine pathways by which the behaviour of melanoma is modified. More sophisticated and rational treatment strategies for this capricious neoplasm might emerge from such studies.

I gratefully acknowledge the technical assistance of Mr L. Ries, Department of Medical Photography. I also want to thank Dr J. G. van Andel and Dr J. H. Mulder for their useful criticism.

### REFERENCES

ALLEN, E. P. (1955) Malignant melanoma; Spontaneous regression after pregnancy. *Br. Med. J.*, ii, 1067.

CLARK, W. H., AINSWORTH, A. M., BERNARDINO, E. A., YANG, C. H., MIHM, M. C. & REED, R. J. (1975) The developmental biology of primary human malignant melanomas. *Semin. Oncol.*, **2**, 83.

CLARK, W. H., FROM, L., BERNARDINO, E. A. & MIHM, M. C. (1969) The histogenesis and biologic behavior of primary human malignant melanomas of the skin. *Cancer Res.*, **29**, 705.

COMIS, R. L. & CARTER, S. K. (1974) Integration of chemotherapy into combined modality therapy for solid tumors. IV. Malignant melanoma. *Cancer Treat. Rev.*, **1**, 285.

FISHER, R. I., NEIFELD, J. P. & LIPPMAN, M. E. (1976) Oestrogen receptors in human malignant melanoma. *Lancet*, ii, 337.

FRIEDMAN, M. A., HOFFMAN, P. G. & JONES, H. W. (1978) The clinical value of hormone receptor assays in malignant disease. *Cancer Treat. Rev.*, **5**, 185.

GEORGE, P. A., FORTNER, J. G. & PACK, G. T. (1960) Melanoma with pregnancy: A report of 115 cases. *Cancer*, **13**, 854.

GILCHRIST, K. W., GILBERT, E., METTER, G. & POWERS, D. (1977) Importance of microscopic vascular invasion in primary cutaneous malignant melanoma. *Surg. Gynecol. Obstet.*, **145**, 559.

HERBST, W. P. (1943) Malignant melanoma of the choroid with extensive metastasis treated by removing secreting tissue of the testicles. *J. Am. Med. Ass.*, **122**, 597.

HERSEY, P., MORGAN, G., STONE, D. E., MCCARTHY, W. H. & MILTON, G. W. (1977) Previous pregnancy as a protective factor against death from melanoma. *Lancet*, i, 451.

MANTEL, N. (1966) Evaluation of survival data and two new rank order statistics arising in its consideration. *Cancer Chemother. Rep.*, **50**, 163.

MCLEOD, G. R., BEARDMORE, G. L., LITTLE, J. H., QUINN, R. L. & DAVIS, N. C. (1971) Results of treatment of 361 patients with malignant melanoma in Queensland. *Med. J. Aust.*, **1**, 1211.

NATHANSON, L., HALL, T. C. & FARBER, S. (1967) Biological aspects of human malignant melanoma. *Cancer*, **20**, 650.

OLSEN, G. (1966) The malignant melanoma of the skin; New theories based on a study of 500 cases *Acta Chir. Scand.* (Suppl.), 365.

PACK, G. T. & SCHARNAGEL, I. M. (1951) The prognosis for malignant melanoma in the pregnant woman. *Cancer*, **4**, 324.

SADOFF, L., WINKLEY, J. & TYSON, S. (1973) Is malignant melanoma an endocrine-dependent tumor? The possible adverse effect of estrogen. *Oncology*, **27**, 244.

SCHMOECKEL, C. & BRAUN-FALCO, O. (1978) Prognostic index in malignant melanoma. *Arch. Dermatol.*, **114**, 871.

SHAW, H. M., MILTON, G W., FARAGO, G. & MCCARTHY, W. H. (1978) Endocrine influences on survival from malignant melanoma. *Cancer*, **42**, 669.

SHIU, M. H., SCHOTTENFELD, D., MACLEAN, B. & FORTNER, J. G. (1976) Adverse effect of pregnancy on melanoma; A reappraisal. *Cancer*, **37**, 181.

SICILIANO, M. J., PERLMUTTER, A. & CLARK, E. (1971) Effect of sex on the development of melanoma in hybrid fish of the genus *Xiphophorus*. *Cancer Res.*, **31**, 725.

SUMNER, W. C. (1953) Spontaneous regression of melanoma; Report of a case. *Cancer*, **6**, 1040.

WEISS, N. S. & FLANNERY, J. T. (1978) The relationship of marital status to survival from melanoma. *Cancer*, **42**, 296.

WHITE, L. P. (1959) Studies on melanoma. II. Sex and survival in human melanoma. *N. Engl. J. Med.* **260**, 789.

WHITE, L. P. (1963) The role of natural resistance in the prognosis of human melanoma. *Ann. N.Y. Acad. Sci.*, **100**, 115.

WHITE, L. P., LINDEN, G., BRESLOW, S. & HARZFELD, L. (1961) Studies on melanoma; The effect of pregnancy on survival in human melanoma. *J. Am. Med. Ass.*, **177**, 235.